# GATA2 regulates the erythropoietin receptor in t(12;21) ALL

**DOI:** 10.18632/oncotarget.19792

**Published:** 2017-08-02

**Authors:** Marie E. Gaine, Daniel J. Sharpe, James S. Smith, Hilary A.A. Colyer, Vivien M. Hodges, Terry R. Lappin, Ken I. Mills

**Affiliations:** ^1^ Centre for Cancer Research and Cell Biology, Queen’s University Belfast, Belfast BT9 7BL, United Kingdom; ^2^ Current/Present address: Department of Molecular Physiology and Biophysics, University of Iowa Carver College of Medicine, Iowa City, IA 52242, USA

**Keywords:** EPOR, GATA2, acute lymphoblastic leukemia, epigenetic, transcriptional regulation

## Abstract

The t(12;21) (p13;q22) chromosomal translocation resulting in the *ETV6/RUNX1* fusion gene is the most frequent structural cytogenetic abnormality in children with acute lymphoblastic leukemia (ALL). The erythropoietin receptor (EPOR), usually associated with erythroid progenitor cells, is highly expressed in *ETV6/RUNX1* positive cases compared to other B-lineage ALL subtypes. Gene expression analysis of a microarray database and direct quantitative analysis of patient samples revealed strong correlation between *EPOR* and *GATA2* expression in ALL, and higher expression of *GATA2* in t(12;21) patients. The mechanism of EPOR regulation was mainly investigated using two B-ALL cell lines: REH, which harbor and express the *ETV6/RUNX1* fusion gene; and NALM-6, which do not. Expression of EPOR was increased in REH cells compared to NALM-6 cells. Moreover, of the six GATA family members only *GATA2* was differentially expressed with substantially higher levels present in REH cells. *GATA2* was shown to bind to the EPOR 5'-UTR in REH, but did not bind in NALM-6 cells. Overexpression of *GATA2* led to an increase in EPOR expression in REH cells only, indicating that GATA2 regulates EPOR but is dependent on the cellular context. Both *EPOR* and *GATA2* are hypomethylated and associated with increased mRNA expression in REH compared to NALM-6 cells. Decitabine treatment effectively reduced methylation of CpG sites in the *GATA2* promoter leading to increased *GATA2* expression in both cell lines. Although Decitabine also reduced an already low level of methylation of the EPOR in NALM-6 cells there was no increase in EPOR expression. Furthermore, *EPOR* and *GATA2* are regulated post-transcriptionally by miR-362 and miR-650, respectively. Overall our data show that EPOR expression in t(12;21) B-ALL cells, is regulated by GATA2 and is mediated through epigenetic, transcriptional and post-transcriptional mechanisms, contingent upon the genetic subtype of the disease.

## INTRODUCTION

The t(12;21) (p13;q22) chromosomal translocation occurs in approximately 25% of cases of childhood B-lineage acute lymphoblastic leukemia (B-ALL). The rearrangement results in the expression of the *ETV6/RUNX1* fusion gene, which leads to increased expression of a number of genes, including the erythropoietin receptor *(EPOR)*, compared to other subtypes of B-ALL [1–4].

ETV6/RUNX1-positive patients have a favorable prognosis but the role of EPOR is poorly understood, as is its mechanism of up-regulation in non-erythroid cells. *In vitro* studies have revealed that erythropoietin (EPO) enhances proliferation of ETV6/RUNX1-positive cells and decreases their sensitivity to prednisone-induced apoptosis [5]. ETV6/RUNX1 directly activates the ectopic expression of functional EPOR *in vitro*; leading to the suggestion that EPOR signaling may contribute to the persistence of covert premalignant clones in pediatric ALL patients with the t(12;21) translocation [6]. In principle the increase in EPOR expression in ETV6/RUNX1 positive cells could arise from complex interactions in the regulation of EPOR involving transcription factors, CpG methylation status of the EPOR promoter or the preponderance of relevant microRNAs (miRNAs).

The expression of EPOR has long been associated with hematopoietic cells committed to the erythroid lineage. Binding of EPO to EPOR on the surface membrane of erythroid progenitors activates the intracellular signaling pathways essential for cell survival, proliferation and differentiation. Over the last decade it has become increasingly clear that EPOR is expressed on numerous normal and malignant cell types. Consequently recombinant EPO treatment is often withheld from cancer patients with anemia, due to the risk of augmenting tumor growth [7].

In developing erythroid cells the expression of EPOR peaks at the proerythroblast stage [8], concurrent with maximal expression of GATA1, an obligatory effector of its transcription [9]. Normally, *GATA1* is weakly expressed in B lymphocytes, therefore this study focused on the possible compensatory role of other members of the GATA family for the transcriptional regulation of EPOR.

The GATA family of basic-helix-loop-helix transcription factors recognizes analogous GATA motifs and has six members, of which GATA1, GATA2 and GATA3 have important functions in hematopoiesis [10]. GATA1 regulates erythropoiesis, megakaryopoiesis and the development of eosinophils and mast cells [11]. GATA2 is essential for the maintenance and proliferation of hematopoietic stem cells and progenitor cells [10, 12]. Evidence that GATA2 can also act as a single lineage-specific transcription factor is provided by *Gata2*^*-/-*^ mice which have a remarkably specific phenotype in which primitive erythropoiesis is strikingly reduced [13].

GATA3 was first identified in a screen for GATA factors in the T cell lineage and plays a key role in early T cell development and the specification of the Th2 subset of T cells [14–16]. A genome-wide germline single nucleotide polymorphism (SNP) analysis identified variants in the GATA3 gene which influence susceptibility to Philadelphia Chromosome-like (Ph-like) ALL and the risk of relapse in childhood ALL [17].

Interplay between GATA factors appears to be a common mechanism for controlling developmental processes [18]. Chromatin occupancy by GATA1 and GATA2 changes during hematopoiesis, leading to lineage-specific differentiation. A recent genome wide analysis demonstrated that GATA1 and GATA2 bind overlapping sets of genes thereby enabling differential regulation of target genes during hematopoiesis [19].

This study examines the mechanisms of EPOR up-regulation through GATA2, including its binding to the *EPOR* promoter, CpG methylation status, and investigation of miRNAs that inhibit *EPOR* and *GATA2* in the two ALL phenotypes.

## RESULTS

**Figure 1:**
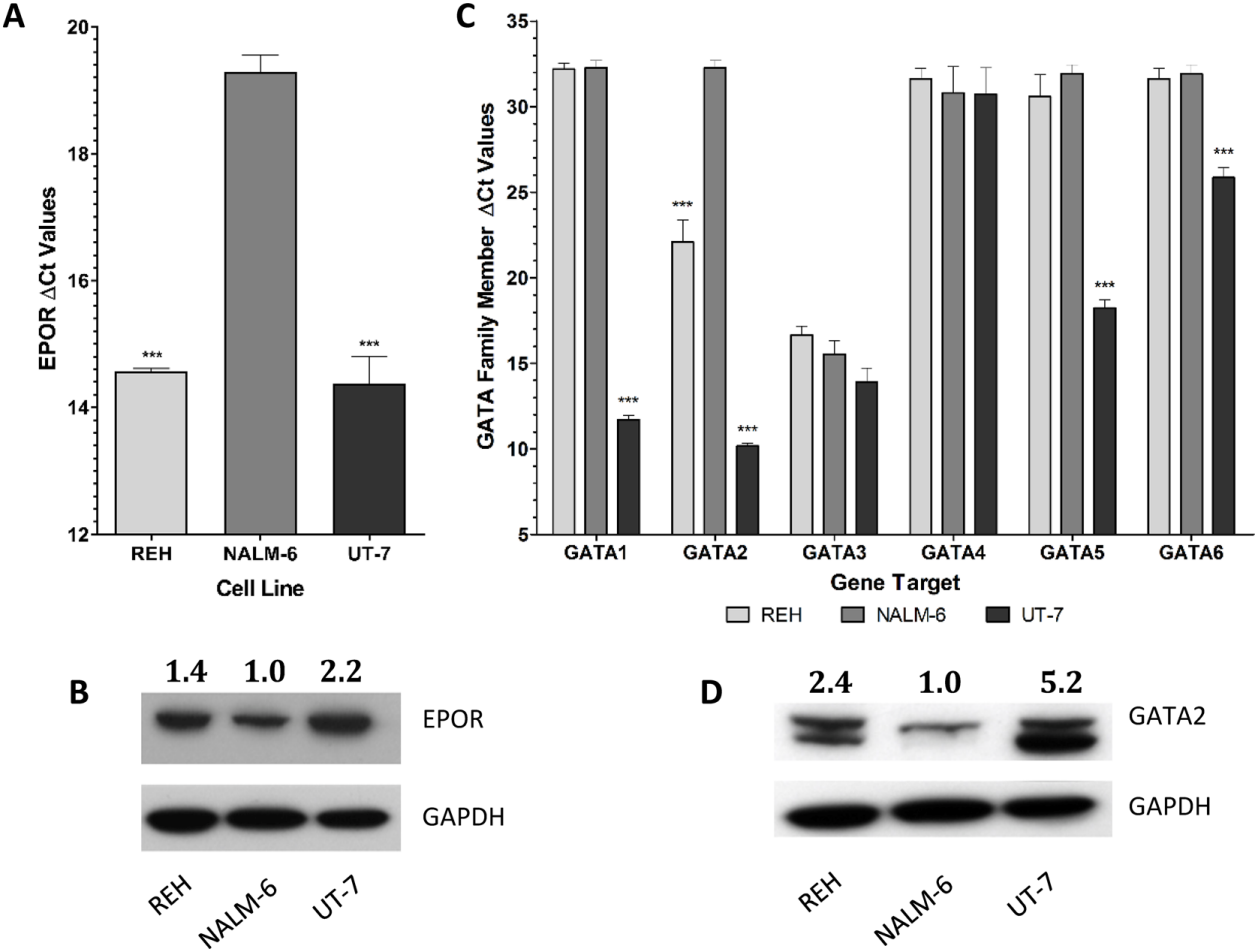
*EPOR* and *GATA* family members are differentially expressed between ETV6/RUNX1 positive and ETV6/RUNX1 negative ALL cell lines **(A)** The expression of *EPOR* was analyzed in REH (ETV6/RUNX1 positive), NALM-6 (ETV6/RUNX1 negative) and UT-7 (*EPOR* positive control) cells in triplicate by Q-PCR. Expression values were corrected to 18S ribosomal RNA levels. Mean corrected Ct values (±SD) are shown and statistical differences to NALM-6 were detected by one-way ANOVA and are indicated by *** (p < 0.001). **(B)** Western blot analysis of EPOR expression in protein extracted from REH, NALM-6 and UT-7 cells. GAPDH was used as a loading control. EPOR expression levels were calculated relative to NALM-6 by densitometric analysis using GAPDH as a normalization factor. **(C)** The expression of each *GATA* family member (*GATA1-6*) was analyzed in REH, NALM-6 and UT-7 cells in triplicate by Q-PCR. Expression values were corrected to 18S ribosomal RNA levels. Mean corrected Ct values (±SD) are shown and statistical differences to NALM-6 were detected by one-way ANOVA and are indicated by *** (p < 0.001). **(D)** Western blot analysis of *GATA2* expression in protein extracted from REH, NALM-6 and UT-7 cells. GAPDH was used as a loading control. *GATA2* expression levels were calculated relative to NALM-6 by densitometric analysis using GAPDH as a normalization factor.

The expression of *EPOR* was determined by Q-PCR in the B-cell progenitor cell lines REH, which is ETV6/RUNX1-positive; NALM-6, which is ETV6/RUNX1 negative and the erythroid cell line, UT-7, known to have high EPOR expression, as a positive control. The high expression of the ETV6/RUNX1 fusion gene in REH cells was confirmed by Q-PCR (Supplementary Figure 1). *EPOR* is highly expressed in REH and UT-7 cells and significantly (p < 0.001) more weakly expressed in NALM-6 cells (Figure 1A). This pattern of expression was confirmed by Western blotting (Figure 1B).

EPOR is tightly regulated in erythroid cells, mainly by GATA1 which is expressed at low levels in B-cell precursors. To investigate whether other members of the GATA family are involved in the expression of EPOR, we evaluated the expression of each GATA family member in the three model cell lines. *GATA4*, *GATA5* and *GATA6* were very weakly expressed in REH and NALM-6 cells, and conversely *GATA3* was highly expressed in both cell lines. However, *GATA2* was significantly (p < 0.001) differentially expressed, with higher levels in REH than in NALM-6 cells (Figure 1C). Western blot analysis indicated that GATA2 protein levels were also higher in REH than NALM-6 cells (Figure 1D). OCI-AML3 was selected as a negative control to check *EPOR* expression in a non-ALL leukemic cell line. Results from the Affymetrix datasets confirm that *EPOR* is substantially higher in UT-7 and REH cells than in NALM-6 and OCI-AML-3 (Supplementary Figure 2).

**Figure 2:**
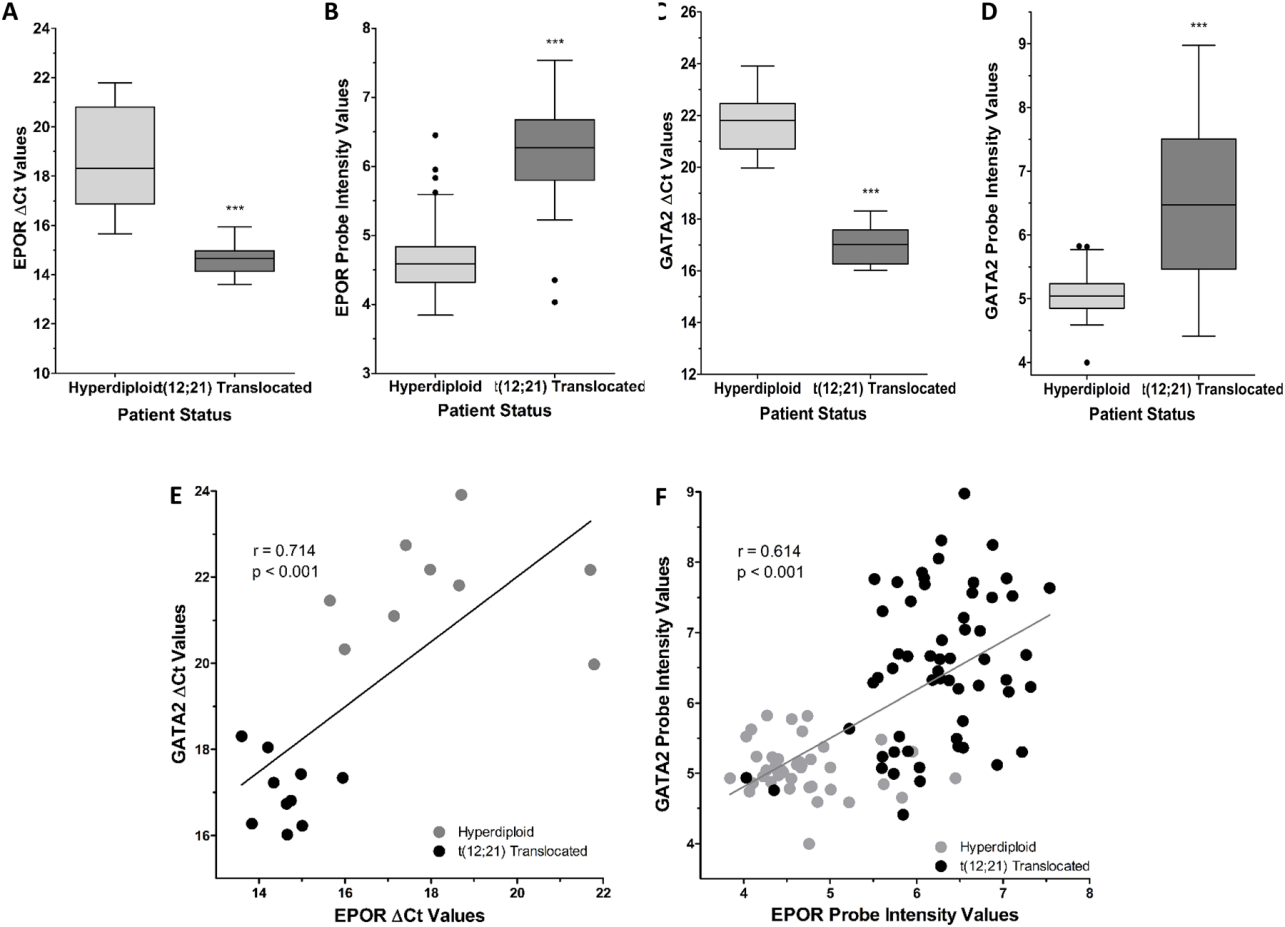
*EPOR* and *GATA2* are differentially expressed between ETV6/RUNX1 positive and ETV6/RUNX1 negative ALL patients **(A)** The expression of *EPOR* was analyzed in hyperdiploid (N=10) and t(12;21) translocated (N=10) ALL patients by Q-PCR. Expression values were corrected to 18S ribosomal RNA levels. **(B)** *EPOR* probe intensities (probe ID: 37986_at) of hyperdiploid (N=40) and t(12;21) translocated (N=58) MILE study ALL patients extracted after normalization of expression files. **(C)** The expression of *GATA2* was analyzed in hyperdiploid (N=9) and t(12;21) translocated (N=10) ALL patients by Q-PCR. Expression values were corrected to 18S ribosomal RNA levels. **(D)** *GATA2* probe intensities (probe ID: 209710_at) of hyperdiploid (N=40) and t(12;21) translocated (N=58) MILE study ALL patients extracted after normalization of expression files. Whiskers indicate Tukey minimum and maximum values; boxes indicate inter-quartile range, with the median marked. Significantly different expression was detected by Student’s t-test with Welch’s Correction and indicated by *** (p < 0.001). **(E)** Correlation between *EPOR* and *GATA2* mRNA expression as measured by Q-PCR in patient samples. *NOTE: High ΔCt values correspond to low gene expression.* Correlation coefficient (r = 0.714) and associated p-value (p < 0.001) were calculated by Pearson’s correlation test. **(F)** Correlation between *EPOR* (probe ID: 37986_at) and *GATA2* (probe ID: 209710_at) intensity values in MILE study patient samples. *NOTE: High Probe Intensity values correspond to high gene expression.* Correlation coefficient (r = 0.614) and associated p-value (p < 0.001) were calculated by Pearson’s correlation test.

The expression of *EPOR* and *GATA2* was further analyzed in RNA extracted from a cohort of pediatric ALL patients, who were either ETV6/RUNX1-positive or negative (hyperdiploid ALL). The expression of the ETV6/RUNX1 fusion gene in these patient samples was confirmed by Q-PCR (Supplementary Figure 3). Figure 2A shows that patients with the fusion gene had, on average, an *EPOR* expression level 3.96 Ct lower (i.e. higher expression) than the ETV6/RUNX1-negative (hyperdiploid) patients; this would represent 15.56-fold higher expression in *EPOR* in ETV6/RUNX1-positive patients (p < 0.001).

High expression of *EPOR* was confirmed by comparing the gene expression intensities between hyperdiploid and ETV6/RUNX1-positive ALL patients in the MILE Study (GEO13159) [20], see Figure 2B and Supplementary Figure 4A. These data revealed that the ETV6/RUNX1-positive ALL patient group had on average 3.2-fold higher *EPOR* expression, compared to the hyperdiploid (ETV6/RUNX1-negative) ALL patient group (p < 0.001); indicating a strong association between ETV6/RUNX1 and *EPOR* expression in B-cell progenitor cells from ALL patients. The *EPOR* was higher in the ETV6/RUNX1-positive ALL patient group than in six other B-ALL subtypes included in the MILE study (Supplementary Figure 4B). *GATA2* expression was also examined in the pediatric ALL cohort, which showed the ETV6/RUNX1-positive group had an average Ct difference of 4.79, representing 27.67-fold higher expression (p < 0.001), Figure 2C.

Figure 2D depicts *GATA2* expression, based on probeset fluorescence intensity, in hyperdiploid or ETV6/RUNX1-positive patients in the MILE Study microarray data (GEO13159) [20]. *GATA2* levels derived from two additional probesets were analyzed (Supplementary Figure 5). The ETV6/RUNX1-positive ALL patient sub-group had on average 2.7-fold higher *GATA2* expression, compared to the ETV6/RUNX1-negative group (p < 0.001); indicating a strong association between ETV6/RUNX1 and *GATA2* expression in B-cell progenitor cells from ALL patients.

A comparison of the expression of *EPOR* and the six *GATA* binding proteins (GATA1–6) between ETV6/RUNX1-positive ALL and hyperdiploid ALL patients in the MILE study is shown in Table 1. The expression of *EPOR*, *GATA2* and *GATA3* are significantly higher in ETV6/RUNX1-positive ALL than in hyperdiploid ALL patients. The lack of *GATA1* expression in the cell line models, and the increased expression of *GATA2* in ETV6/RUNX1-positive ALL compared to hyperdiploid ALL patients suggest that *EPOR* expression may be regulated by *GATA2* in this type of B-cell leukemia.

**Table 1:** Expression of *EPOR* and *GATA* binding proteins in t(12;21) ALL and hyperdiploid ALL from the MILE Study

	Probeset ID	Gene symbol	Gene title	ALL with t(12;21) vs. ALL hyperdiploid		
				p Value	Fold change	Higher in
Probeset E1	37986_at	EPOR	erythropoietin receptor	2.98E-21	2.95025	ALL with t(12;21)
Probeset E2	215054_at	EPOR	erythropoietin receptor	1.74E-20	2.8498	ALL with t(12;21)
Probeset E3	209962_at	EPOR	erythropoietin receptor	8.69E-18	3.35594	ALL with t(12;21)
Probeset E4	209963_s_at	EPOR	erythropoietin receptor	2.08E-10	1.899	ALL with t(12;21)
Probeset E5	396_f_at	EPOR	erythropoietin receptor	5.63E-05	1.30265	ALL with t(12;21)
Probeset E6	216999_at	EPOR	erythropoietin receptor	0.183499	-1.02559	
Probeset G1.1	1555590_a_at	GATA1	GATA binding protein 1	0.558919	-1.03024	
Probeset G1.2	210446_at	GATA1	GATA binding protein 1	0.939326	1.00597	
Probeset G2.1	209710_at	GATA2	GATA binding protein 2	2.47E-12	2.68299	ALL with t(12;21)
Probeset G2.2	210358_x_at	GATA2	GATA binding protein 2	1.27E-07	1.28291	ALL with t(12;21)
Probeset G2.3	207954_at	GATA2	GATA binding protein 2	0.460004	1.01988	
Probeset G3.1	209604_s_at	GATA3	GATA binding protein 3	3.70E-05	2.15746	ALL with t(12;21)
Probeset G3.2	209602_s_at	GATA3	GATA binding protein 3	0.00157696	1.45584	ALL with t(12;21)
Probeset G3.3	209603_at	GATA3	GATA binding protein 3	0.0263834	1.19443	ALL with t(12;21)
Probeset G4.1	230855_at	GATA4	GATA binding protein 4	0.11158	1.06661	
Probeset G4.2	1570276_a_at	GATA4	GATA binding protein 4	0.243658	-1.02736	
Probeset G4.3	205517_at	GATA4	GATA binding protein 4	0.267507	-1.02403	
Probeset G4.4	243692_at	GATA4	GATA binding protein 4	0.317532	-1.03456	
Probeset G4.5	1553131_a_at	GATA4	GATA binding protein 4	0.794327	-1.00928	
Probeset G5.1	238095_at	GATA5	GATA binding protein 5	0.591853	-1.01384	
Probeset G5.2	238197_at	GATA5	GATA binding protein 5	0.872993	1.00297	
Probeset G6.1	229282_at	GATA6	GATA binding protein 6	0.169085	-1.06536	
Probeset G6.2	210002_at	GATA6	GATA binding protein 6	0.20102	-1.03821	

The relationship between *EPOR* and *GATA2* mRNA levels in individual patient samples showed a strong positive correlation between *EPOR* and *GATA2*, (R = 0.714, p < 0.001) in the pediatric ALL patients with ETV6/RUNX1 (Figure 2E). Analysis of the MILE gene expression data also revealed a strong positive correlation between *EPOR* and *GATA2* mRNA levels (R = 0.614, p < 0.001) (Figure 2F). Interestingly 2 of the 58 ETV6/RUNX1 positive cases in Figure 2F show very low *EPOR* expression and very low *GATA2*, consistent with the idea that GATA2 regulates EPOR. These observations indicate that the strict relationship between the high expression of *EPOR* and *GATA2* in ETV6/RUNX1 patients is not maintained in a minority cases and may reflect phenotypic diversity among the 58 patients in this subgroup.

Correlations between each of the *EPOR* and *GATA2* probesets from the MILE study are detailed in Supplementary Table 3. This provides further evidence in support of an interaction between *EPOR* and *GATA2* in the presence of ETV6/RUNX1.

To investigate whether EPOR is transcriptionally regulated by GATA2, REH and NALM-6 cells were transfected with the pENTR221-GATA2 over-expression vector. Over-expression of *GATA2* mRNA was confirmed by Q-PCR in both cell lines 72 hr after transfection. In NALM-6 cells, which do not normally express *GATA2*, there was a dramatic increase in *GATA2* as indicated by an approximately 15,000-fold change (p < 0.001), whilst in REH cells, a ≈ 4,000-fold change in expression was observed (p < 0.001) (Supplementary Figure 6A). There was no significant change in *EPOR* expression in NALM-6 cells, but there was a significant increase in *EPOR* expression (Fold change = 13.4, p < 0.001) in REH cells (Supplementary Figure 6B). Thus, overexpression of *GATA2* causes an increase in *EPOR* expression in REH cells, but not in NALM-6, suggesting that additional factors necessary for EPOR regulation by GATA2 are only present in REH cells.

**Figure 3:**
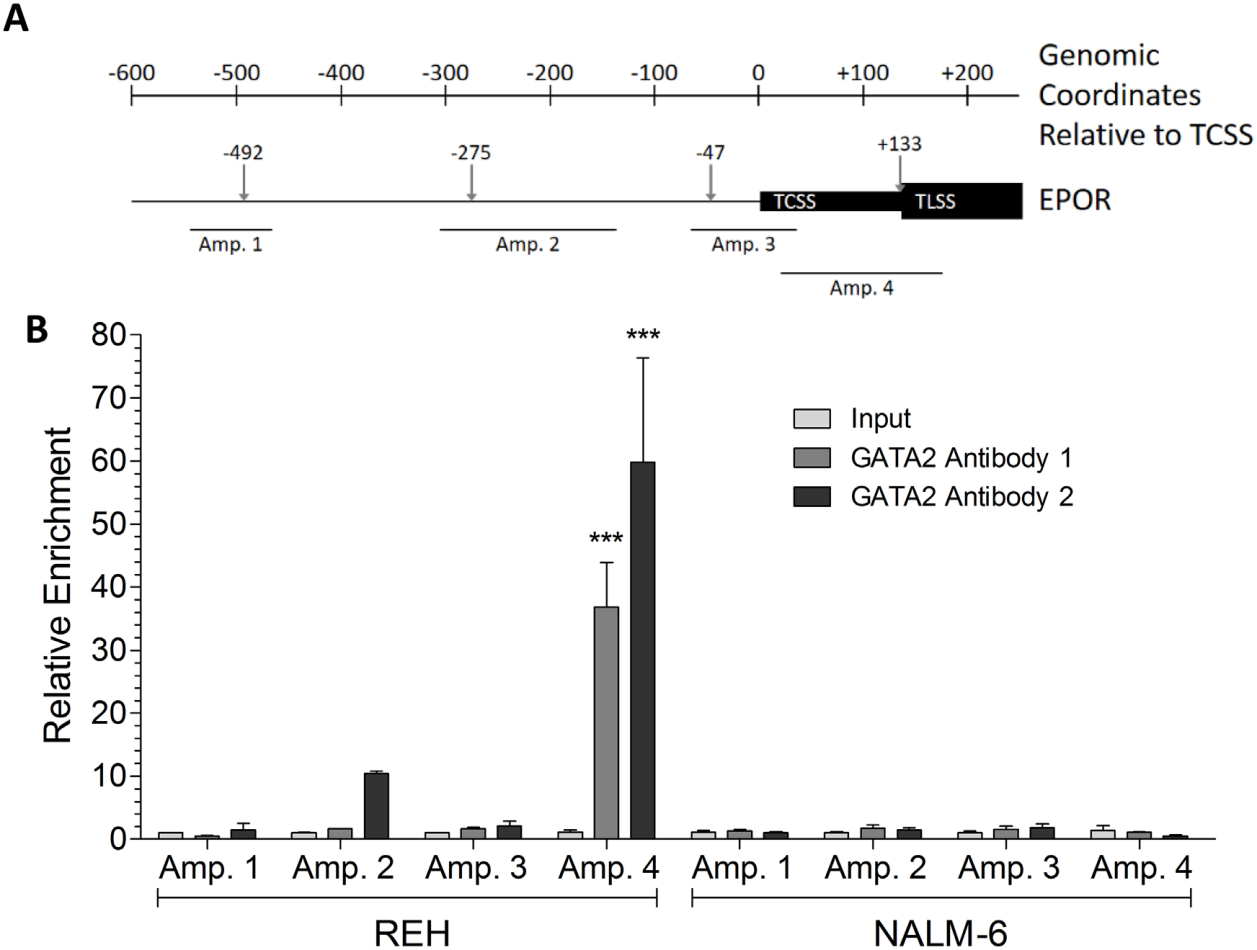
GATA2 binds to the 5′ UTR region of the *EPOR* gene in REH, but not NALM-6, cells **(A)** Schematic of the *EPOR* genomic locus showing the relative positions of predicted GATA2 binding sites (↓), the *EPOR* transcription start site (TCSS), the *EPOR* translation start site (TLSS) and the amplicon targets in ChIP experiments. All genomic coordinates are given relative to the TCSS. **(B)** ChIP assays were performed on formaldehyde-fixed chromatin prepared from REH and NALM-6 cells. Enrichment of GATA2 binding to *EPOR* 5′ DNA was determined by comparison to a non-specific binding region and input chromatin controls. Two independent GATA2 antibodies were used. GATA2 binding enrichment was assessed at four genomic loci (Amplicon 1-4). Significant enrichments were detected by two-way ANOVA with Bonferroni’s post-hoc test and are indicated by *** (p < 0.001).

Chromatin immunoprecipitation (ChIP) analysis utilizing both murine and rabbit antibodies revealed that GATA2 binds to the EPOR promoter of the genomic locus at the most 3´ GATA2 site within the 5´-UTR in REH cells, but GATA2 does not bind to the EPOR promoter in NALM-6 cells (Figure 3B). This suggested a difference in the 5´-UTR of NALM-6 cells that inhibits GATA2 binding to the EPOR promoter. An alternative possibility, that the low level of GATA2 in NALM-6 cells is insufficient to transactivate the EPOR promoter was excluded by the data obtained by forced expression of *GATA2* (Supplementary Figure 6).

**Figure 4:**
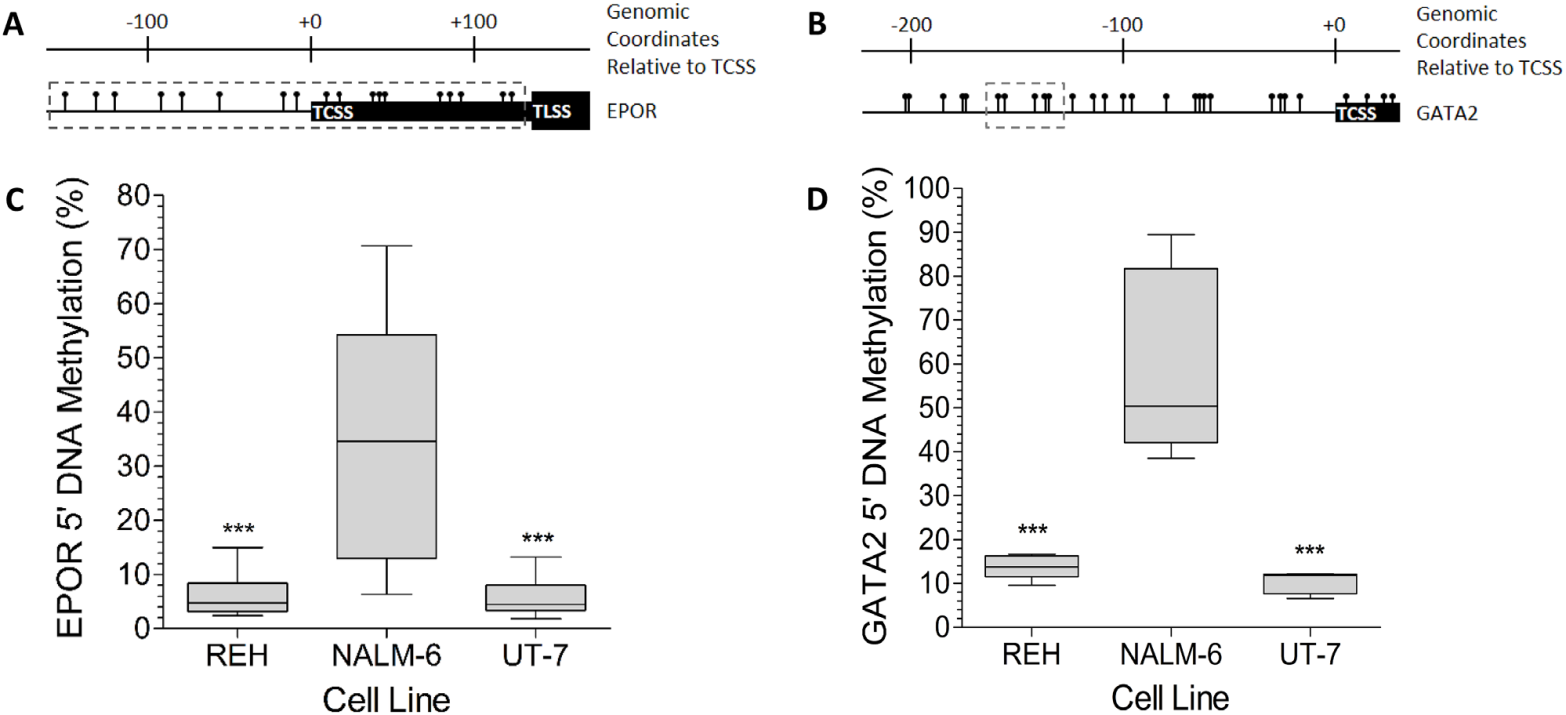
The *EPOR* and *GATA2* 5′ DNA is highly methylated in NALM-6, but not in REH cells **(A)** Schematic of the *EPOR* genomic locus showing the relative positions of CpG dinucleotides (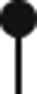), the *EPOR* transcription start site (TCSS), the *EPOR* translation start site (TLSS) and the CpG sites included in the pyrosequencing assay (grey dashed box). All genomic coordinates are given relative to the TCSS. **(B)** Schematic of the *GATA2* genomic locus showing the relative positions of CpG dinucleotides (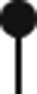), the *GATA2* transcription start site (TCSS) and the CpG sites included in the pyrosequencing assay (grey dashed box). **(C)** *EPOR* 5′ DNA specific pyrosequencing assays were performed on bisulphite converted DNA prepared from REH, NALM-6 and UT-7 cells. DNA methylation was assessed at 18 CpG sites in triplicate. Whiskers indicate Tukey minimum and maximum CpG methylation values; boxes indicate inter-quartile range, with the median marked. Significant enrichments were detected by one-way ANOVA and are indicated by *** (p < 0.001). **(D)** *GATA2* 5′ DNA specific pyrosequencing assays were performed on bisulphite converted DNA prepared from REH, NALM-6 and UT-7 cells. DNA methylation was assessed at 5 CpG sites in triplicate. Whiskers indicate Tukey minimum and maximum CpG methylation values; boxes indicate inter-quartile range, with the median marked. Significant enrichments were detected by one-way ANOVA and are indicated by *** (p < 0.001).

The methylation status of eighteen individual CpG sites in the *EPOR* promoter (Figure 4A) of the three cell lines was determined by pyrosequencing. The mean methylation levels were 35.8%, 5.1% and 4.9% for the NALM-6, REH, and UT-7 cells respectively (Figure 4C). REH and UT-7 cells have comparable levels of methylation, less than 20%, at all of the CpG sites (Supplementary Figure 7A). In contrast, some of the CpG sites in the NALM-6 cells showed substantially higher methylation, up to 70%, with 16 sites showing significantly higher (p < 0.001) levels than the corresponding CpG sites in REH and UT-7 cells. Pyrosequencing of five CpG sites in the *GATA2* upstream region (Figure 4B) revealed mean methylation levels of 59.6% for NALM-6, 13.8% for REH, and 10.28% for UT-7 cells (Figure 4D); significantly higher methylation was found at all sites in the NALM-6 than in either REH or UT-7 cells (p < 0.001; Supplementary Figure 7B). The lower expression of both *EPOR* and *GATA2* seen in NALM-6 cells, compared to REH and UT-7 cells, is consistent with the concept that epigenetic silencing of genes is frequently associated with hypermethylation of the promoter region.

We investigated the effect of Decitabine on the *EPOR* promoter in NALM-6 cells, see Supplementary Figure 8A. Of the 18 CpG sites analyzed, 15 showed statistically significant decreases in methylation after treatment with Decitabine in the range 50 to 500 nM. CpG sites 7, 8 and 18 have low DNA methylation status which did not change upon Decitabine treatment. No significant changes in methylation status of the *EPOR* promoter were found in REH cells after treatment with Decitabine, see Supplementary Figure 9A.

**Figure 5:**
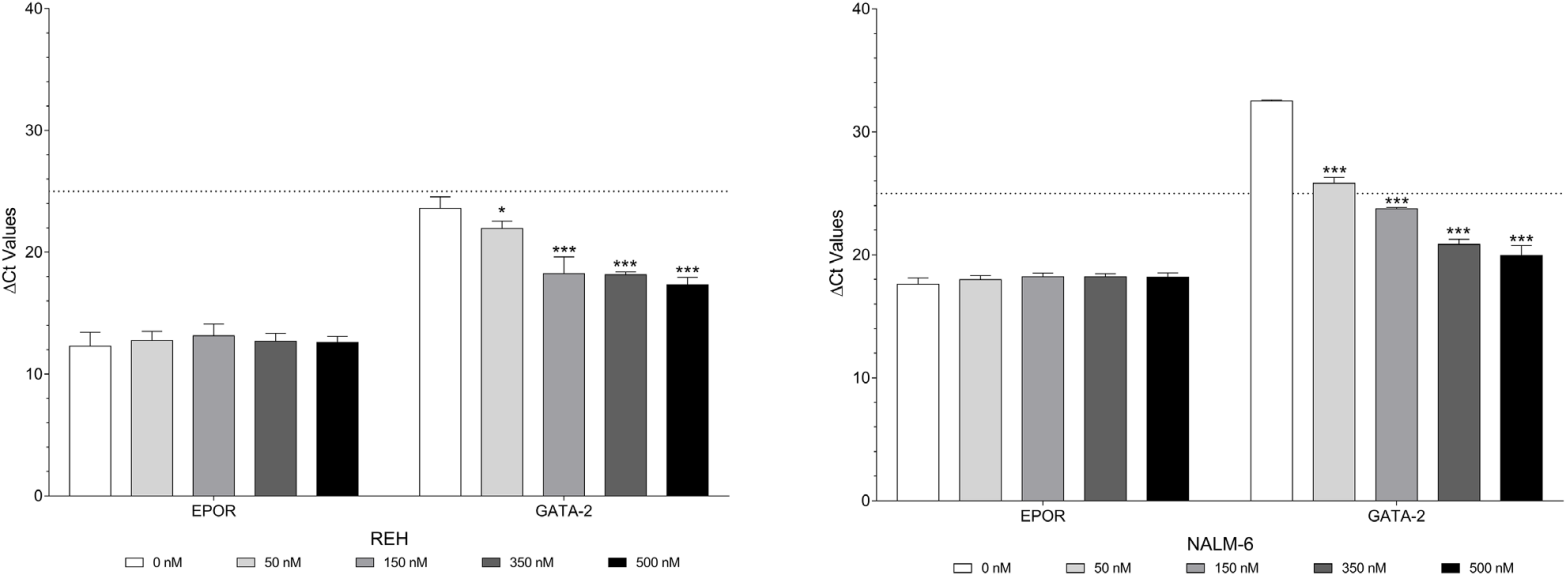
Decitabine causes demethylation of both *EPOR* and *GATA2* but only increases expression of *GATA2* *EPOR* and *GATA2* expression in REH and NALM-6 cells after treatment with 50 to 500 nM Decitabine. Expression values were corrected to 18S ribosomal RNA levels. Mean corrected Ct values (±SD) are shown. Statistical differences compared to the control (0 nM Decitabine) are indicated by * (p < 0.05), ** (p < 0.01), or *** (p < 0.001) were calculated with the two-way ANOVA with Holm Sidak correction.

The effect of Decitabine on the *GATA2* promoter methylation status in NALM-6 cells is shown in Supplementary Figure 8B. Decitabine in the range 50 to 500 nM caused significant reductions in percentage methylation at all five of the CpG sites. The effect of Decitabine on *GATA2* promoter methylation status was less marked in REH cells (Supplementary Figure 9B). Decitabine in the range 50 to 500 nM caused significant upregulation of *GATA2* expression in both REH and NALM-6 cells. In contrast the changes in *EPOR* expression were minimal in both cell lines, see Figure 5.

**Figure 6:**
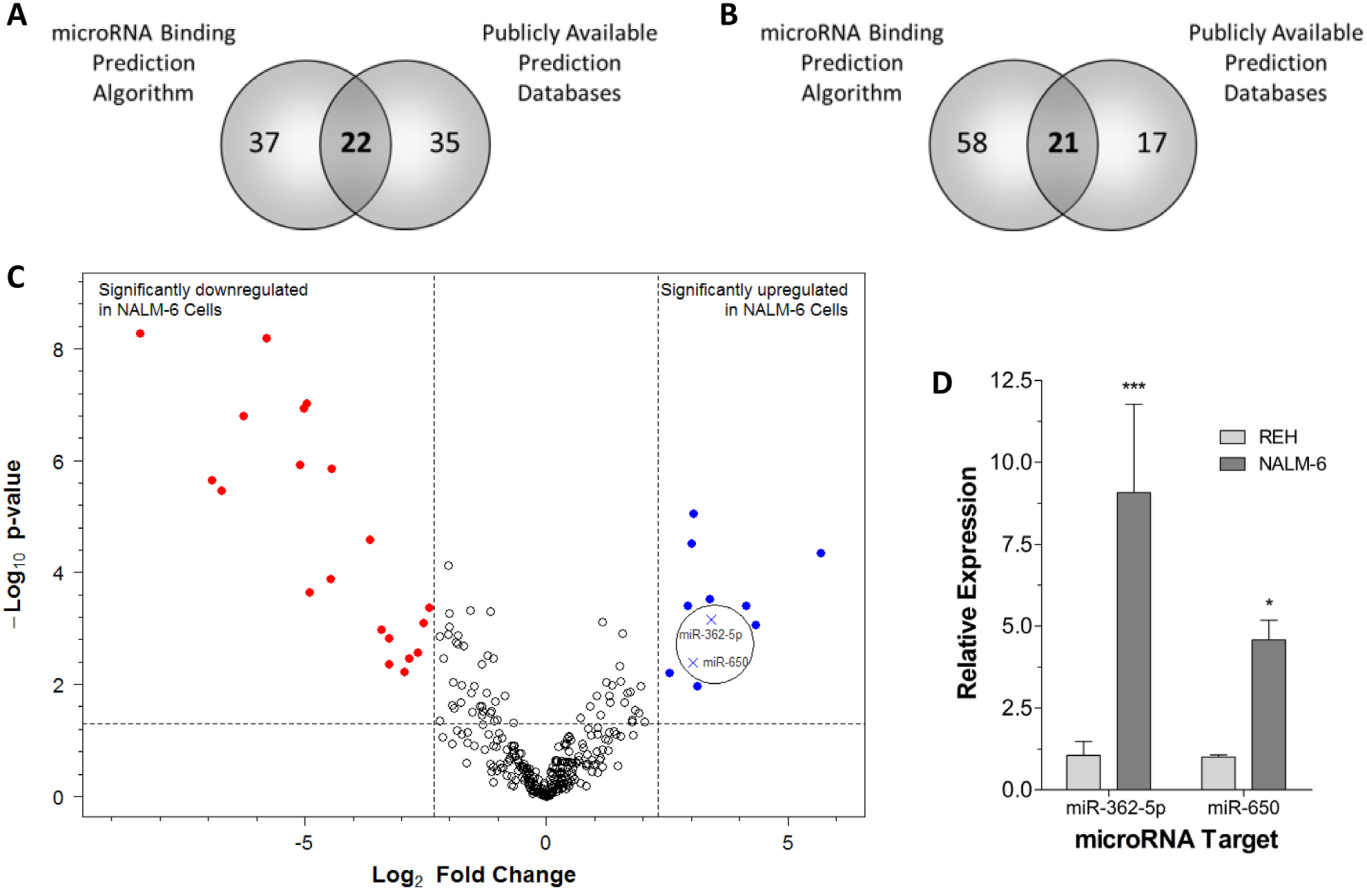
Expression of microRNAs predicted to target EPOR and GATA2 are increased in NALM-6 cells **(A)** Venn diagram showing consensus between an *in silico* microRNA targeting algorithm [45] and publicly available prediction databases. MicroRNAs predicted to target *EPOR* were selected and overlapped. **(B)** Venn diagram showing consensus between an *in silico* microRNA targeting algorithm [45] and publicly available prediction databases. MicroRNAs predicted to target GATA2 were selected and overlapped. **(C)** Volcano plot of the differential expression of microRNAs between REH and NALM-6 cells and the associated nominal p-value. The expression of 670 microRNAs was analyzed in REH and NALM-6 cells in triplicate by Q-PCR using multiplex assays. Expression values were corrected to the mean RNU6 and RNU44 levels. Nominal p-values associated with the fold differences compared to NALM-6 were determined using the Bioconductor package ‘limma’. Selection criteria for significantly different expression were an absolute fold change ≥5 and a nominal p-value < 0.05. **(D)** The differential expression of selected microRNAs (miR-362-5p and miR-650) was validated in REH and NALM-6 cells in triplicate by Q-PCR using single microRNA assays. Expression values were corrected to the mean RNU6 and RNU44 levels. Mean relative expression levels (±SD) compared to REH are shown and statistical differences to REH were detected by two-way ANOVA and are indicated by * (p < 0.05) or *** (p < 0.001).

MicroRNAs are a class of epigenetic regulatory molecules that act at the post-transcriptional level to repress target genes by inhibition of translation and destabilization of mRNA [21]. Twenty-two miRNAs were predicted to target EPOR based on data collated from the Pareto Front predictive algorithm, the publicly available ‘miRecords’ target prediction meta-database, and a strongly negative “Probability of Interaction by Target Accessibility” (PITA) score (Figure 6A, Supplementary Methods, and Supplementary Table 4). Of these only miR-362-5p showed an overlap with those significantly up-regulated in NALM-6 cells (labeled in Figure 6C). Similarly, 21 miRNAs were predicted to target GATA2 based on the same criteria (Figure 6B and Supplementary Table 4). Of these only miR-650 was significantly up-regulated (labeled in Figure 6C). To investigate the miRNA profiles in REH and NALM-6 cells Taqman® microRNA arrays were performed in triplicate. The arrays allow simultaneous analysis of 667 miRNAs and showed that 11 miRNAs were significantly up-regulated and 20 miRNAs were significantly down-regulated in NALM-6 cells compared to REH cells (Figure 6C). Expression data obtained from microRNA array analysis were validated by single assay Q-PCR which confirmed that the expression of both miR-362 and miR-650 are higher in NALM-6 than in REH cells (Figure 6D).

**Figure 7:**
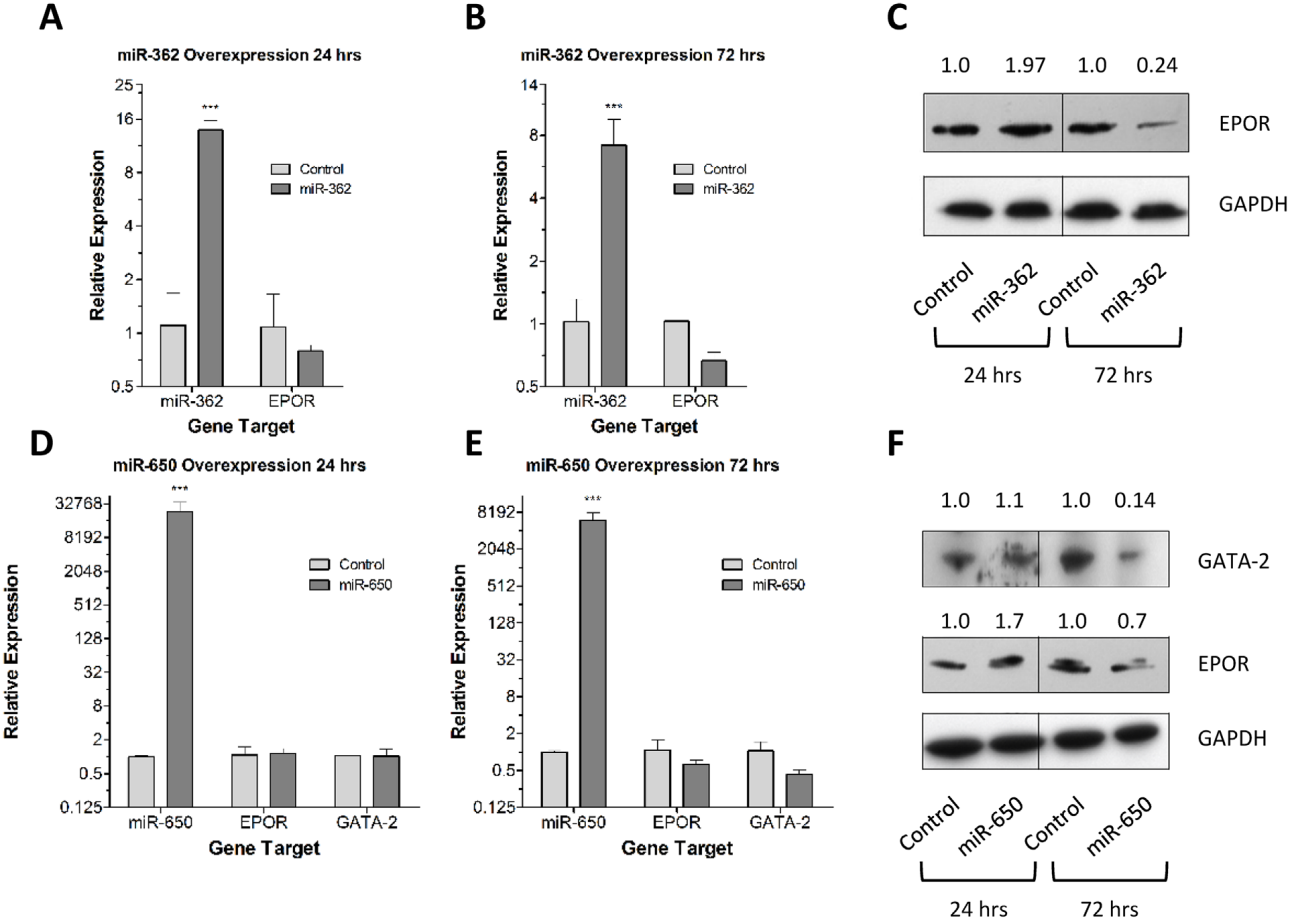
Forced expression of miR-362-5p and miR-650 reduces *EPOR* and GATA2 expression **(A)** The expression of miR-362-5p and *EPOR* were analyzed in REH cells in triplicate by Q-PCR 24 hr post-transfection with a miR-362-5p expression vector. Expression values were corrected to 18S ribosomal RNA levels. Mean relative expression levels (±SD) compared to empty vector controls are shown and statistical differences to control were detected by one-way ANOVA and are indicated by *** (p < 0.001). **(B)** The expression of miR-362-5p and *EPOR* were analyzed in REH cells in triplicate by Q-PCR 72 hr post-transfection with a miR-362-5p expression vector. Expression values were corrected to 18S ribosomal RNA levels. Mean relative expression levels (±SD) compared to empty vector controls are shown and statistical differences to control were detected by one-way ANOVA and are indicated by *** (p < 0.001). **(C)** Western blot analysis of EPOR expression in protein extracted from REH cells 24 hr. and 72 hr. post-transfection with a miR-362-5p expression vector. GAPDH was used as a loading control. EPOR expression levels were calculated relative to htR (EV control) by densitometric analysis using GAPDH as a normalization factor. **(D)** The expression of miR-650, *EPOR* and *GATA2* were analyzed in REH cells in triplicate by Q-PCR 24 hr. post-transfection with a miR-650 mimetic oligo. Expression values were corrected to 18S ribosomal RNA levels. Mean relative expression levels (±SD) compared to scrambled oligo controls are shown and statistical differences to control were detected by one-way ANOVA and are indicated by *** (p < 0.001). **(E)** The expression of miR-650, *EPOR* and *GATA2* were analyzed in REH cells in triplicate by Q-PCR 72 hr. post-transfection with a miR-650 mimetic oligo. Expression values were corrected to 18S ribosomal RNA levels. Mean relative expression levels (±SD) compared to scrambled oligo controls are shown and statistical differences to control were detected by one-way ANOVA and are indicated by *** (p < 0.001). **(F)** Western blot analysis of EPOR and GATA2 expression in protein extracted from REH cells 24 hr. and 72 hr. post-transfection with a miR-650 mimetic oligo. GAPDH was used as a loading control. *EPOR* and *GATA2* expression levels were calculated relative to scrambled oligo control by densitometric analysis using GAPDH as a normalization factor.

miR-362-5p, predicted to target EPOR, was over-expressed in REH cells, confirmed by Q-PCR, (Figure 7A & 7B) and a modest, but not significant, decrease in *EPOR* mRNA expression was observed after 72 hr. However, a four-fold decrease in EPOR protein levels was observed at 72 hr post-transfection with miR-362-5p (Figure 7C). These data suggest that miR-362-5p can regulate *EPOR* expression in REH cells. The overexpression of miR-650, predicted to target *GATA2*, in REH cells was confirmed by Q-PCR (Figure 7D & 7E). Both *GATA2* and *EPOR* mRNA and protein expression were examined at 24 hr and 72 hr post-transfection. A decrease in *GATA2* mRNA was observed after 72 hr, but did not reach statistical significance; however, a seven-fold decrease in GATA2 protein was found after 72 hr. *EPOR* expression of both mRNA and protein decreased by approximately 30% at 72 hr post-transfection with miR-650. Overall our data suggest that miR-650 regulates GATA2 protein expression and as a consequence EPOR expression whilst miR-362-5p regulates *EPOR* expression in REH cells.

## DISCUSSION

The *EPOR* promoter is unusual because it lacks a TATA box in the core promoter region, but it does contain functional SP-1 and GATA binding sequences. SP-1 is a ubiquitous transcription factor unlikely to drive lineage-specific expression of *EPOR*. Since GATA1 is an obligatory transcription factor for *EPOR* expression in erythroid cells, but is expressed at extremely low levels in B cell progenitors, it was important to determine if other members of the GATA family might contribute to the increased expression of *EPOR* found in ETV6/RUNX1-positive cells. Of the six GATA family members only *GATA2* was found to be differentially expressed with substantially higher levels present in REH cells, i.e. those with the t(12;21) translocation.

GATA2 has a pivotal role in hematopoietic stem and progenitor cell development and its expression decreases with differentiation implying that GATA2 is necessary to maintain pluripotency [22]. The data indicate that *GATA2* is a regulator of the *EPOR* gene in t(12;21) B cell ALL. This is supported by pediatric t(12;21) ALL patients and ETV6/RUNX1-positive cells having both highly expressed levels of *EPOR* and *GATA2* with a strong positive correlation. This trend was confirmed by *in silico* analysis of the MILE expression profiling data (GSE13159) which showed that *GATA2* was more highly expressed and exhibits a stronger correlation with *EPOR* expression in ALL patients with the t(12;21) translocation.

High *GATA2* expression is a poor prognostic marker in pediatric myeloid leukemia. Following chemotherapy *GATA2* was found to be normalized in patients in complete remission but remained high in those with resistant disease [23]. Recently it has been reported that reduction of *GATA2* by shRNA or the inhibitor K7174 sensitizes KG1a acute myeloid leukemia cells to chemotherapy [24], suggesting that suppression of *GATA2* expression or inhibition of its transcriptional activity may have potential as an ancillary therapy in AML.

*GATA2* is overexpressed in acute myeloid leukemia (AML) and loss-of-function mutations have been causally linked to immunodeficiency associated with the myelodysplastic syndromes (MDS), suggesting that an appropriate level of GATA2 activity is a prerequisite for normal hematopoiesis [25]. The role of GATA2 as a tumor suppressor remains to be defined. Vicente and colleagues have proposed a transcriptional network involved in the emergence of hematopoietic stem cells in which GATA2, FLI1 and SCL form a complex that is recruited to the RUNX1 enhancer, activating its transcription [25]. Mutations in any of the constituent genes may perturb this regulatory system and alter the phenotype of the daughter cells.

A mechanistic link between RAS, which harbors activating mutations in 30% of all human cancers, and GATA2 has recently been uncovered [26]. Downstream from RAS, MAPK p38 phosphorylates a number of residues in GATA2, the most critical of which is S192, leading to GATA2 transcriptional activity. In this context GATA3 has also been found to promote cycling of hematopoietic stem cells and to restrict the long-term reconstitutive potential downstream of p38 in stress-induced hematopoiesis [27]. *GATA2* transcription is regulated by several transcription factors including ETS1, BMP4, NOTCH1, PU.1 and EVI1 (reviewed by Vicente [25]). The regulatory elements also contain GATA motifs so that *GATA2* expression is regulated both by itself and by GATA1.

The UT-7/Epo sub-line was derived through continuous long-term culture of the human leukemic cell line UT-7 in the presence of EPO [28]. The higher levels of GATA1 mRNA found in UT-7/Epo compared to UT-7 cells indicated that the cells of the sub-line were committed to the erythroid lineage. Apart from the special case of UT-7/Epo cells there is little evidence that EPO can stimulate leukemic cell proliferation, though in principle survival and proliferation of leukemic cells could be enhanced by presence of functional EPO receptors.

Beyond the hematopoietic compartment EPO can induce GATA3 and GATA4 leading to the up-regulation of *EPOR* expression. Thus EPO stimulation of myoblasts causes up-regulation of GATA3, GATA4 and another basic-helix-loop-helix factor, TAL1. In turn, these transcription factors up-regulate *EPOR* expression in the myoblast [29, 30]. Moreover *EPOR* expression in neural NT2 cells can be *trans*-activated by GATA3, which is required for brain development [31]. These observations highlight the ability of different GATA factors to *trans*-activate the erythropoietin receptor in the context of cellular phenotype.

The different genetic sub-types of ALL can be characterized by distinct DNA methylation signatures that demonstrate significant correlation with expression profiles [32]. *EPOR* was one of sixteen genes found to be specifically hypo-methylated in the t(12;21) subtype and associated with increased mRNA expression [33]. Higher methylation of one CpG site in *GATA2* was found in sub-types of ALL, compared to controls, but no difference was found between the t(12;21) and hyperdiploid sub-types [34].

In the present work, GATA2 was found to bind to *EPOR* downstream of the transcriptional start site in REH cells but not in NALM-6 cells and overexpression of *GATA2* increased *EPOR* production only in REH cells, supporting the suggestion that other regulatory mechanisms are involved. DNA methylation analysis of *EPOR* showed approximately 7-fold higher levels of methylation in the ETV6/RUNX1-negative NALM-6 cells compared to REH and UT-7. Methylation of 15 of 18 CpGs located 5' of the *EPOR* promoter was higher in NALM-6 than in REH cells, but one of the three exceptions was the most proximal CpG to the GATA binding site identified by ChIP. Similarly the *GATA2* promoter region of NALM-6 showed 4- and 5-fold higher levels of methylation compared to REH and UT-7 cells respectively.

Decitabine caused significant demethylation of 15 of the 18 CpG sites analyzed on the *EPOR* promoter in NALM-6 cells, but this did not lead to increased *EPOR* expression. Similarly, Decitabine reduced an already low level of methylation of the *EPOR* promoter in REH cells, but did not cause increased *EPOR* expression. Decitabine caused significant reductions in methylation of all five CpG sites analyzed in the *GATA2* promoter in both NALM-6 and REH cells and led to increased *GATA2* expression in both cell lines.

MicroRNAs are short non-coding RNA molecules of 19-25 nucleotides with the capacity to regulate genes post-transcriptionally by silencing specific gene expression and inhibiting protein translation [21]. miRNAs are involved in a wide range of biological processes, and can function as instructive determinants of cell fate during ontogeny. miRNAs are frequently dysregulated in human cancers, including leukemia: some exert tumor suppressive effects while others promote cancer progression by enhancing tumor growth, angiogenesis, invasion and immune evasion [35]. A global reduction in miRNA expression has been found in cancer and different types of leukemia [36].

Deletion models show that miRNAs are essential regulators of hematopoietic stem and progenitor cell survival, differentiation and function [37]. miRNAs can function as instructive determinants of cell fate during ontogeny. miR-142-3p is absolutely conserved across vertebrates and modulates the maturation of myeloid, erythroid and T cell lineages. Recent work indicates that miR-142-3p functions as a master regulator of HSC specification in *Xenopus* development by controlling a growth regulatory network which includes *tgfbr1, fli1* and *gata2* [38].

miRNAs which were significantly differentially expressed between REH and NALM-6 cells were compared to those predicted by miRecords and Pareto Front analysis to identify those likely to cause down-regulation of *EPOR* and *GATA2*. Overexpression of miR-362 caused down-regulation of EPOR protein levels. Similarly, overexpression of miR-650 caused down-regulation of both *GATA2* and *EPOR*, providing further evidence for a functional relationship between GATA2 and EPOR.

The exact roles of mir-362 and mir-650 in the growth regulatory networks of hematopoietic cells and in the genetic sub-types of leukemic cells remain to be defined. Whereas both microRNAs are differentially expressed in the REH and NALM-6 cell line models, neither was found to be differentially expressed when seven subtypes of ALL were compared in a comprehensive study of 81 childhood cases using 397 microRNAs [39].

It is of interest that mir-362 is up-regulated by EPO in UT-7 cells [40], which suggests that mir-362 forms part of a feedback loop to downregulate *EPOR* following EPO-induced activation of the receptor. miR-362-5p has also been reported to act as an oncomiR by down-regulating GADD45α, which in turn activated the JNK1/2 and P38 signaling in CML patient samples [41]. miR-650 has been reported to target proteins important for B cell proliferation and survival and to affect the biology of chronic lymphocytic leukemia [42].

The GATA gene family activates and represses target genes through multiple mechanisms in a context-dependent manner (reviewed by Bresnick [18]). The present work has provided evidence that GATA2 can up-regulate *EPOR* in ETV6/RUNX1 positive sub-type of B-ALL. This up-regulation occurs via a complex series of epigenetic, transcriptional, and post-transcriptional events which are context-dependent. However, the elucidation of these relationships between GATA2 and EPOR in other subtypes of B-ALL will be required to determine their clinical and therapeutic potential.

## MATERIALS AND METHODS

### Cell lines and patient samples

Details of the cell lines and culture conditions are shown in Supplementary Methods and Supplementary Table 1. Gene expression data were extracted from the Microarray Innnovations in LEukemia (MILE) Study [20], Gene Expression Omnibus accession number GSE13159.

### RNA and DNA extraction

Total RNA and genomic DNA were isolated using the RNeasy Mini Kit and the DNeasy Blood and Tissue Kit (Qiagen). Purity and concentration were evaluated using a NanoDrop 1000 Spectrophotometer (Thermo Scientific). RNA was prepared from bone marrow of ALL patients [43].

### MicroRNA extraction and array analysis

Total RNA was extracted using mirVana™ miRNA Isolation kits (Ambion). The Taqman® MicroRNA Reverse Transcription kit and Megaplex™ RT Primers (Applied Biosystems) were used to synthesize ss-cDNA. MicroRNAs were quantified using Taqman® Human microRNA array cards (Applied Biosystems) and Partek-GSS. Target prediction was performed using miRecords [44] and Pareto Front analysis software [45]. Validation assays were performed using Taqman® MicroRNA Assays.

### Quantitative real-time PCR (Q-PCR)

Cell-line cDNA was generated using M-MLV Reverse Transcriptase (Invitrogen). Patient cDNA was generated using Superscript III First Strand Synthesis System (Invitrogen). Q-PCR was performed on the 7900HT Fast Real-Time PCR System (Applied Biosystems). *EPOR* and *GATA1-6* expression was measured using Taqman probes (Applied Biosystems). ETV6/RUNX1 fusion expression was measured using SYBR Green primers (Roche; available on request).

### Pyrosequencing (Qiagen)

Primers were designed using PyroMark Assay Design 2.0 software (Supplementary Table 2). Genomic DNA was deaminated using an EpiTect Bisulfite Kit and complete conversion checked by Calponin PCR [46]. Regions of interest were amplified using the PyroMark PCR kit before pyrosequencing on a Q24 Instrument (Qiagen).

### Decitabine treatment of cells

To 3x10^6^ REH or NALM-6 cells 5-Aza-2'-deoxycytidine (Decitabine; Sigma-Aldrich) was added to give final concentrations of 50 nM, 150 nM, 350 nM and 500 nM. Water served as the untreated control. After each 48 hour interval the cells were pelleted and resuspended in 3 ml of media and freshly prepared Decitabine. After 7 days, the period required for four doublings for each cell line, pellets prepared from 1.5 ml of each cell suspension were used for RNA and DNA extraction.

### Protein extraction and western blotting

Protein, extracted using RIPA buffer and quantitated using the Pierce BCA Protein Assay, was resolved by SDS-PAGE (12%). Protein was transferred to nitrocellulose membrane (Sigma), incubated with anti-EPOR, anti-GATA2, or anti-GAPDH antibodies overnight at 4°C and visualized using Advansta WesternBright ECL (MyBio). Band density was analyzed using Li-Cor Image Studio Lite version 4.0. The EPOR antibody was a murine monoclonal obtained from Abnova cat no: H00002057-M01. The GATA2 antibody was a rabbit polyclonal obtained from Abcam cat no: ab22849.

### Chromatin immunoprecipitation

Chromatin immunoprecipitation was performed as previously described [47]. Briefly, chromatin was isolated from formaldehyde-cross-linked REH and NALM-6 cells, sheared by sonication and immunoprecipitated overnight with anti-GATA2 antibodies bound to magnetic beads (Invitrogen). Isolated complexes were washed before reversal of the DNA-protein cross-linking and DNA purification by QIAquick columns (Qiagen). DNA was subjected to Q-PCR analysis with gene promoter or non-specific region primers to evaluate promoter DNA enrichment.

### Over-expression of *GATA2*, *miR-650* (pre-*miR-650*) and *miR-362*

Cells were seeded 24 hr. before transfection with 2 μg of negative control (pENTR221-β-glucuronidase), *GATA2* (pENTR221-GATA2) or GFP vector using an Amaxa Nucleofector I Device. Nucleofector Kit T and Kit R (Lonza) were used to transfect NALM-6 and REH respectively. At 72 hr. samples were taken for Q-PCR or western blot. REH cells were transfected with 30 nM pre-miR-650 (Ambion) or FAM-labeled Pre-mir Negative Control #1 (Applied Biosystems). Identical methods were used to transfect with miR-362 or control htr vectors [48] obtained from the Human miRNA Library (Source BioScience).

## SUPPLEMENTARY MATERIALS FIGURES AND TABLES




