# Supplementary material for: GATA2 regulates the erythropoietin receptor in t(12;21) ALL
Source: Oncotarget. 2017 Aug 2;8(39):66061–74. doi: 10.18632/oncotarget.19792 (PMC5630392; doi:10.18632/oncotarget.19792)
Supplement: Supplementary file 1 [file oncotarget-08-66061-s001.pdf]

GATA2 regulates the erythropoietin receptor in t(12;21) ALL

SUPPLEMENTARY MATERIALS

Cell culture and patient samples

The REH (ETV6/RUNX1 positive human B-cell precursor leukemia) and NALM-6 (ETV6/RUNX1 negative human B-cell precursor leukemia) cell lines were purchased from DSMZ GmbH (Braunschweig, Germany) and maintained in RPMI media (including L-glutamine; PAA) containing 100 U/ml penicillin (Invitrogen), 100 µg/ml streptomycin (Invitrogen) and 10% heat-inactivated fetal calf serum (HI-FCS; Invitrogen). The UT-7 cell line served as a positive control due to its known high expression of *EPOR* [1]. UT-7 cells were maintained in MEMα media (including L-Glutamine and nucleosides; PAA) containing 100 U/ml penicillin, 100 µg/ml streptomycin, 10% HI-FCS and 10 ng/ml human GM-CSF (PeproTech). Patient sample RNA, were obtained from the bone marrow of children between the ages of 2.4 to 13.3 years of age with either ETV6/RUNX1 or hyperdiploid diagnosis as previously described [2].

The MILE study

The Microarray Innovations in Leukaemia (MILE) Study was formed in 2005 and provides access to over 3,334 gene expression profiles of sixteen classes of acute and chronic leukemia, MDS and normal bone marrow all established using gold standard methods to ensure accuracy and reproducibility [3]. Data mining was performed on Gene Expression Omnibus data set GSE13159 using the Partek Genomics Suite software. Data pre-processing included a summarization and quantile normalization step to generate probe set level signal intensities for each microarray experiment and was performed as previously described [4].

Probability of interaction by target accessibility (PITA)

PITA [5] involves first searching the 3′UTR for perfect complementary seed sites and then applies a thermodynamic model to each of these sites. Sites for a specific miRNA are then combined, which eliminates the possibility that sites can be bound simultaneously as this requires more information than is available. Filters concerning conservation and mRNAs can be applied by the user. The final PITA score denotes an energy score, $\Delta\Delta G$, which is equal to the difference between the energy gained by binding of the miRNA to the target and the energy required to make the site accessible. This is based on the energetic cost to freeing base-pairing interactions with mRNA secondary structure.

REFERENCES

1. Chretien S, Moreau-Gachelin F, Apiou F, Courtois G, Mayeux P, Dutrillaux B, Cartron JP, Gisselbrecht S, Lacombe C. Putative oncogenic role of the erythropoietin receptor in murine and human leukemia cells. Blood. 1994; 83:1813-21.
2. Cario G, Stanulla M, Fine BM, Teuffel O, Neuhoff NV, Schrauder A, Flohr T, Schäfer BW, Bartram CR, Welte K, Schlegelberger B, Schrappe M. Distinct gene expression profiles determine molecular treatment response in childhood acute lymphoblastic leukemia. Blood. 2005; 105:821-6.
3. Kohlmann A, Kipps TJ, Rassenti LZ, Downing JR, Shurtleff SA, Mills KI, Gilkes AF, Hofmann WK, Basso G, Dell'orto MC, Foà R, Chiaretti S, De Vos J, et al. An international standardization programme towards the application of gene expression in routine leukemia diagnostics: the Microarray Innovations in Leukemia study prephase. Br J Haematol. 2008; 142:802-7.
4. Liu WM, Li R, Sun JZ, Wang J, Tsai J, Wen W, Kohlmann A, Williams PM. PQN and DQN: algorithms for expression microarrays. J Theor Biol. 2006; 243:273-8.
5. http://genie.weizmann.ac.il/pubs/mir07/mir07_prediction.html

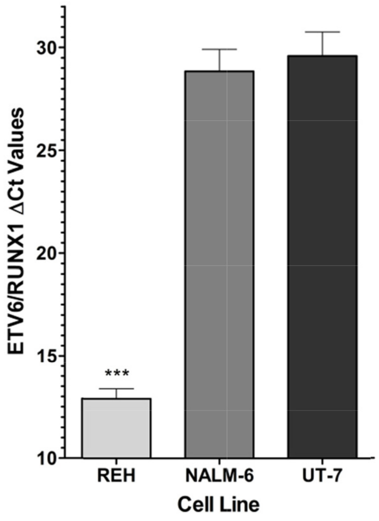

Supplementary Figure 1: *ETV6/RUNX1* fusion gene expression in cell lines. The expression of *ETV6/RUNX1* was analyzed in REH, NALM-6 and UT-7 cells in triplicate by real-time quantitative PCR. Expression values were corrected to 18S ribosomal RNA levels. Mean corrected Ct values (±SD) are shown and statistical differences to NALM-6 were detected by one-way ANOVA and are indicated by *** ($p < 0.001$).

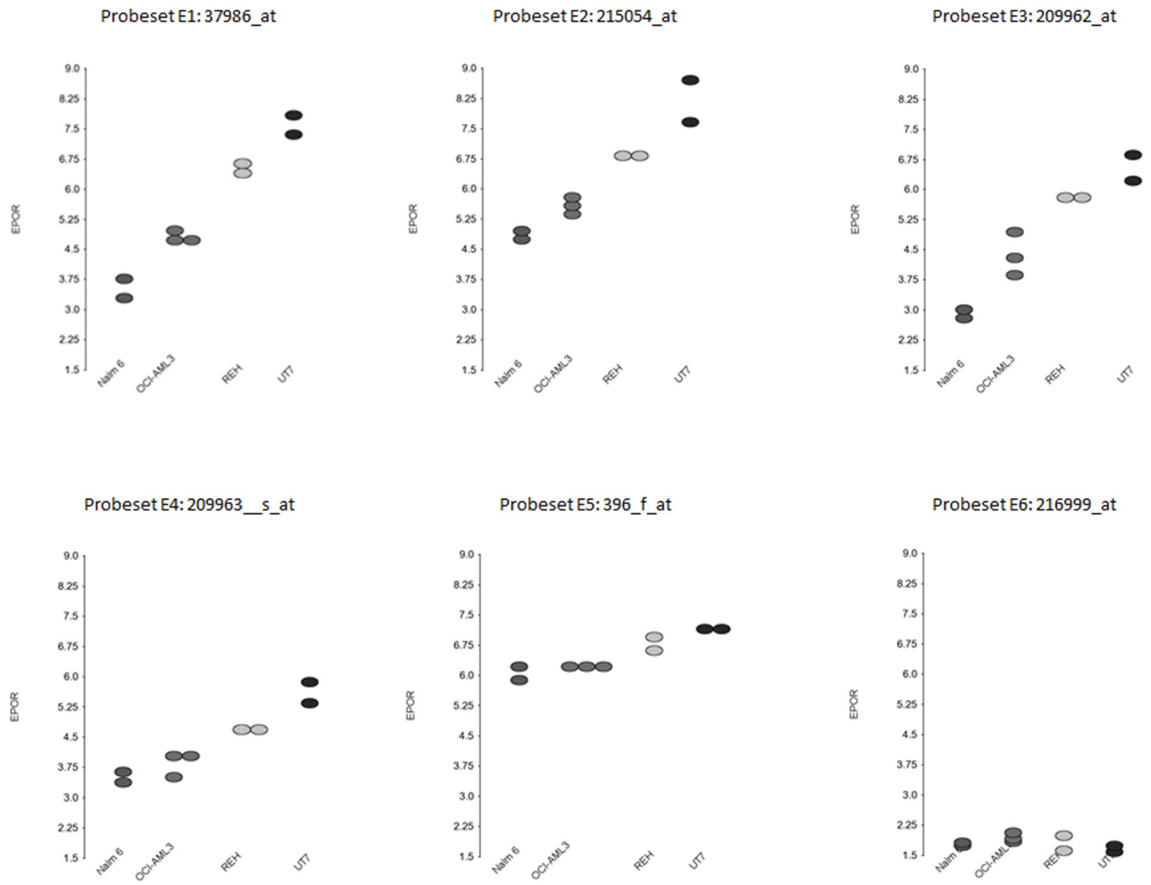

Supplementary Figure 2: *EPOR* Expression in OCI-AML3, NALM-6, REH and UT7 cell lines. *EPOR* expression (log2 intensity) levels obtained from Affymetrix U133Plus2 gene arrays for four cell lines across the six *EPOR* probesets. The cell lines used were NALM-6 and REH cells representing ALL, UT-7 cells which have a high level of EPOR expression due to a mutation and OCI-AML3 cells that are an AML cell line. As expected NALM-6 always showed lower or equivalent expression to the OCI-AML3 cells, whilst UT-7 cells were always the highest. REH cells showed between 0.8 - 3 log2 intensity values higher than NALM-6; representing 1.74 – 8 fold higher expression in REH compared to NALM-6. It should be noted that probeset 216999_at shows a different and consistently low pattern of expression indicating that it is not a robust probeset particularly as it shows a low result for *EPOR* in UT-7, the cell line that is archetypal for EPOR function and high expression.

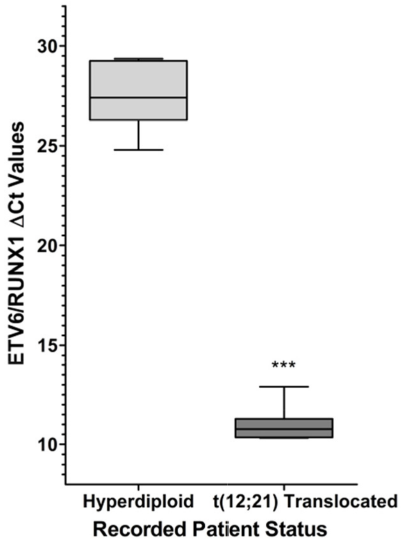

Supplementary Figure 3: *ETV6/RUNX1* expression in patient samples. The expression of *ETV6/RUNX1* was analyzed in patient samples. Expression values were corrected to 18S ribosomal RNA levels. Mean corrected Ct values (±SD) are shown and statistical differences to hyperdiploid patients were detected by Student's t-test with Welch's Correction and indicated by *** ($p < 0.001$).

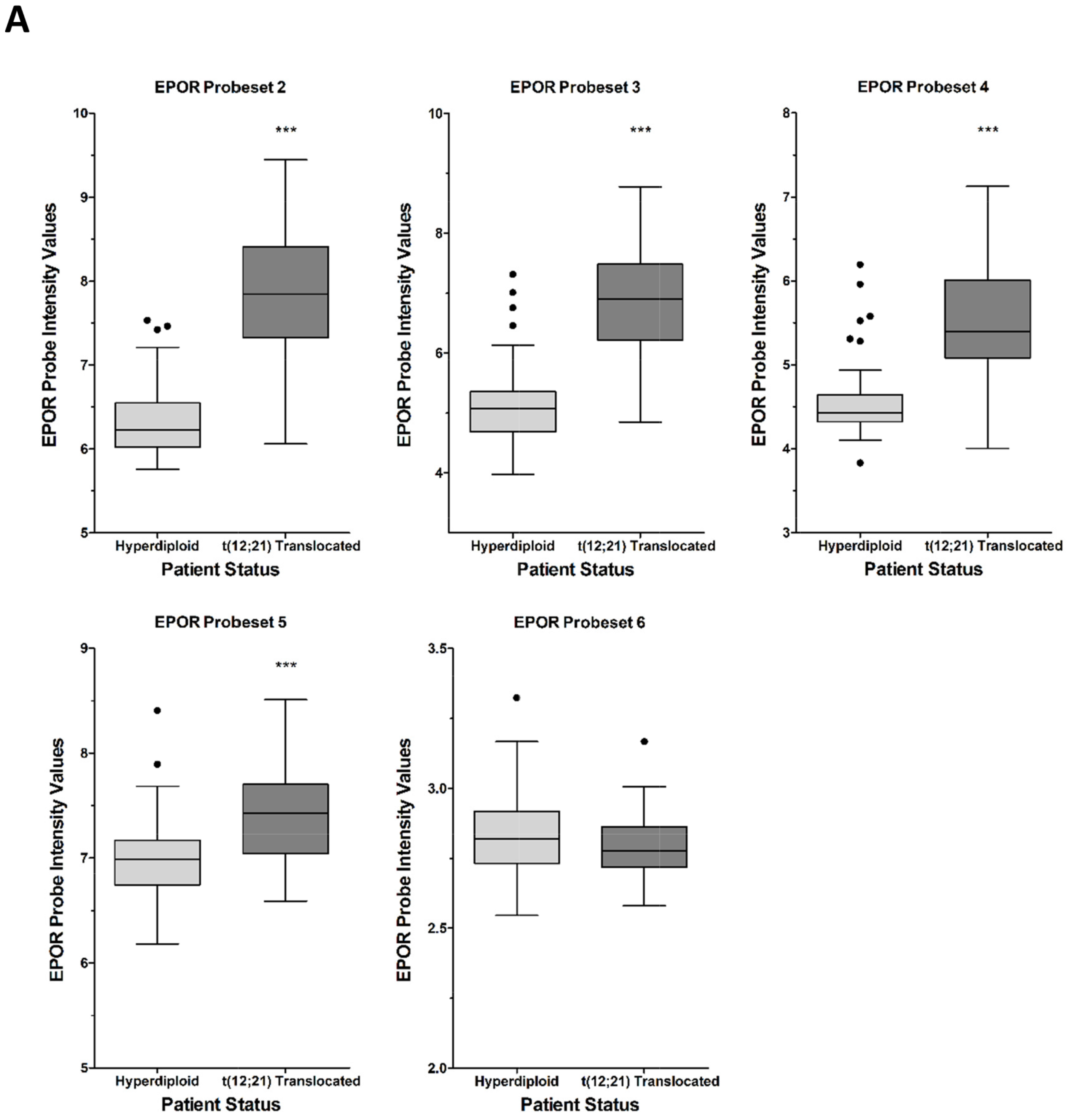
A
EPOR Probeset 2
EPOR Probeset 3
EPOR Probeset 4
EPOR Probeset 5
EPOR Probeset 6
EPOR Probe Intensity Values
***
Hyperdiploid
t(12;21) Translocated
Patient Status

(*Continued*)

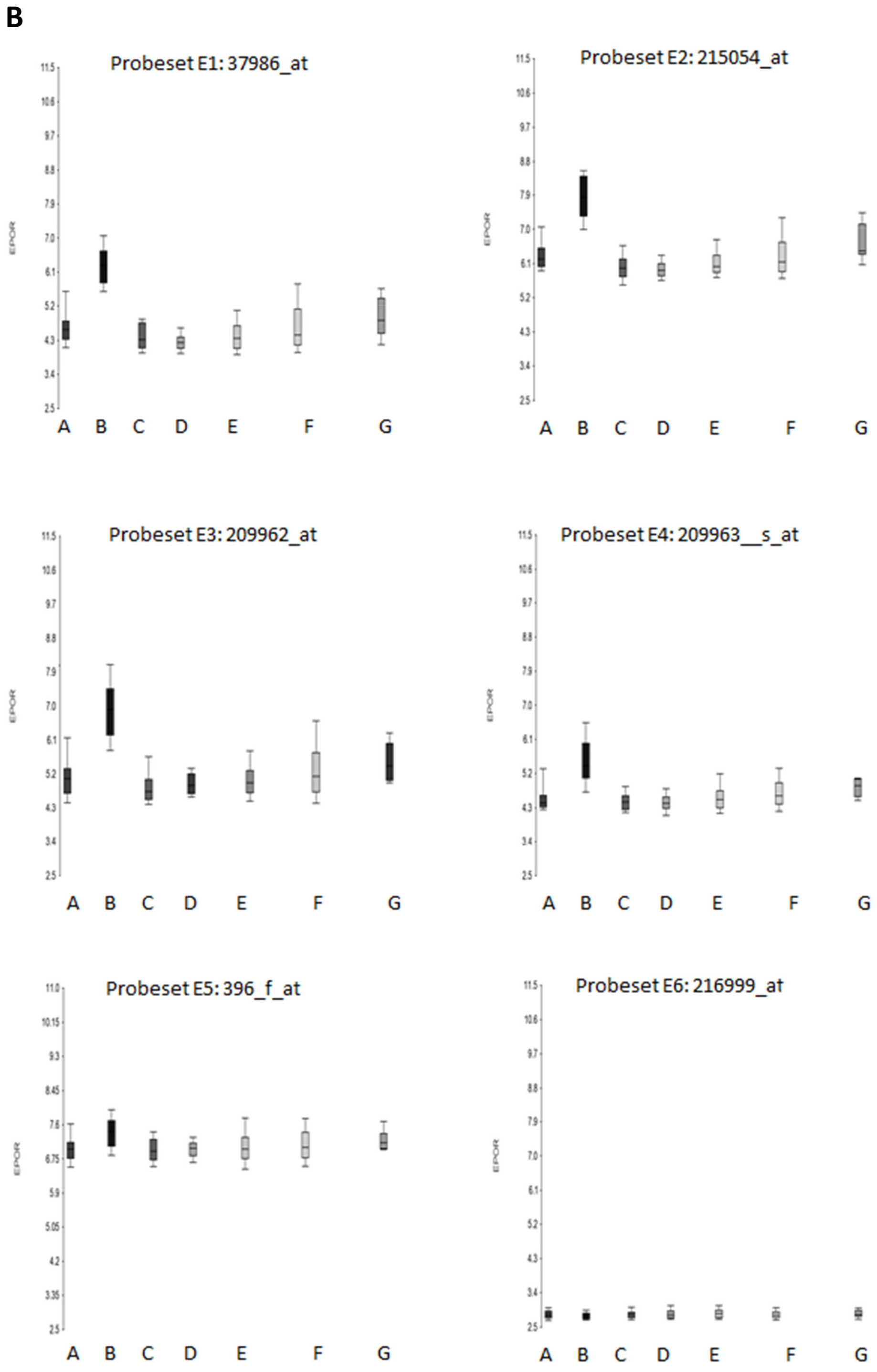

Supplementary Figure 4: *EPOR* probe intensities MILE study patients. **(A)** *EPOR* expression level (log2 intensity) of hyperdiploid (N=40) and t(12;21) translocated (N=58) ALL patients in the MILE study (GEO13159) [26] for probesets 2 – 6. **(B)** *EPOR* expression (log2 intensity) levels obtained from Affymetrix U133Plus2 gene arrays for the six *EPOR* probesets across seven B-ALL subtypes included in the MILE study [26]. (A) ALL with hyper-diploid karyotype (40 patients); (B) ALL with t(12;21) (58 patients); **(C)** ALL with t(1;19) (36 patients); **(D)** Pro-B-ALL with t(11q23)/MLL (70 patients); **(E)** c-ALL/Pre-B-ALL with t(9;22) (122 patients); **(F)** c-ALL/Pre-B-ALL without t(9;22) (237 patients); and **(G)** mature B-ALL with t(8;14) (13 patients). It should be noted that probeset 216999_ shows a different and consistently low pattern of expression indicating that it is not a robust probeset.

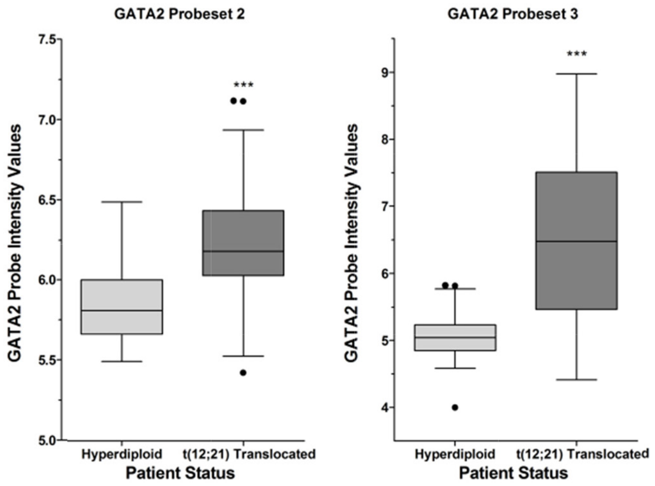

Supplementary Figure 5: *GATA2* probe intensities from hyperdiploid and t(12;21) translocated MILE study patients. *GATA2* probe intensities of hyperdiploid (N=40) and t(12;21) translocated (N=58) ALL patients extracted after normalization of MILE study (GEO13159) expression files.

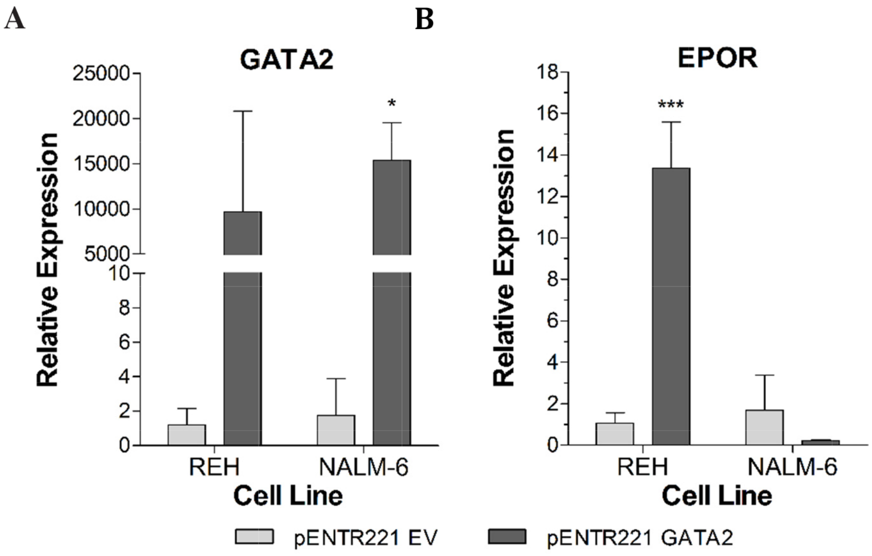

Supplementary Figure 6: Forced expression of *GATA2* increases *EPOR* expression in REH, but not NALM-6 cells. (A) Q-PCR analysis confirming the overexpression of *GATA2* in REH and NALM-6 cells 72 hr post-transfection. Mean relative expression levels (±SD) compared to Empty Vector (EV) controls are shown and statistical differences to EV were detected by one-way ANOVA and are indicated by * ($p < 0.05$). **(B)** Q-PCR analysis of *EPOR* expression 72 hr post-transfection with EV or *GATA2* expression vector. Mean relative expression levels (±SD) compared to Empty Vector (EV) controls are shown and statistical differences to EV were detected by one-way ANOVA and are indicated by *** ($p < 0.001$).

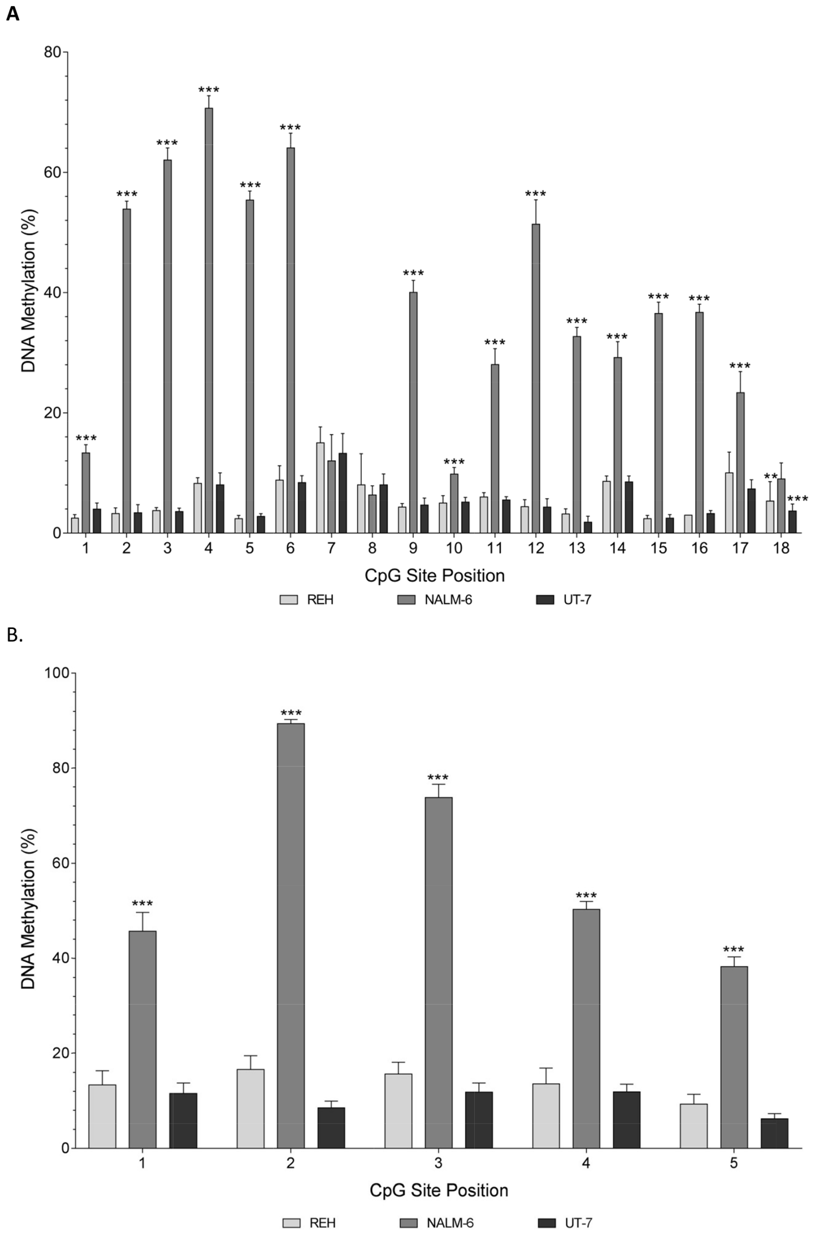

Supplementary Figure 7: DNA methylation of the *EPOR* and *GATA2* genes in cell lines. DNA methylation levels of eighteen CpG sites 5′ of the *EPOR* gene **(A)** and of the five CpG sites 5′ of the *GATA2* gene **(B)** in REH, NALM-6 and UT-7 cells. Mean percentage DNA methylation for each CpG site (± SD) is shown. Statistical differences compared to NALM-6 cells are indicated by * ($p < 0.05$) or *** ($p < 0.001$) were calculated with the two-way ANOVA with Holm-Sidak correction.

A

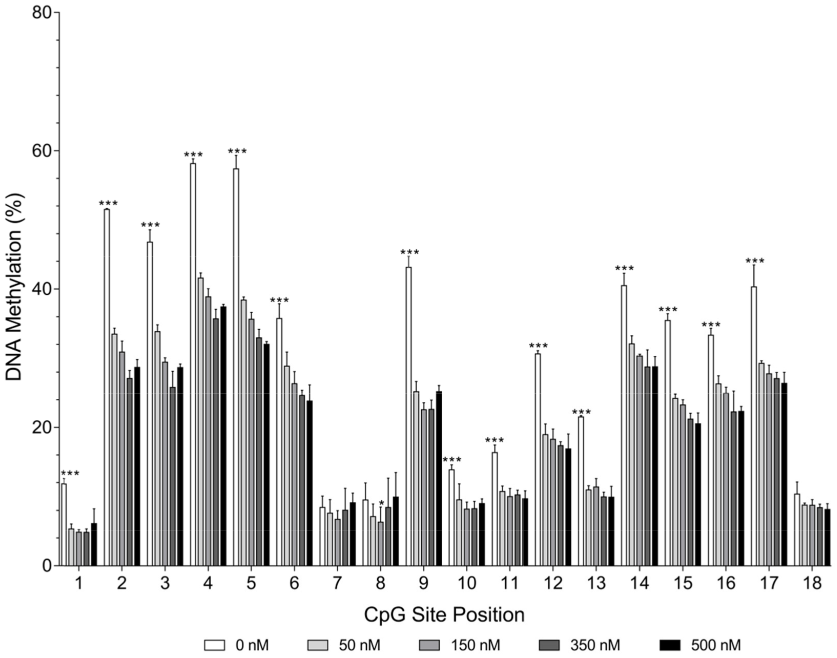

B

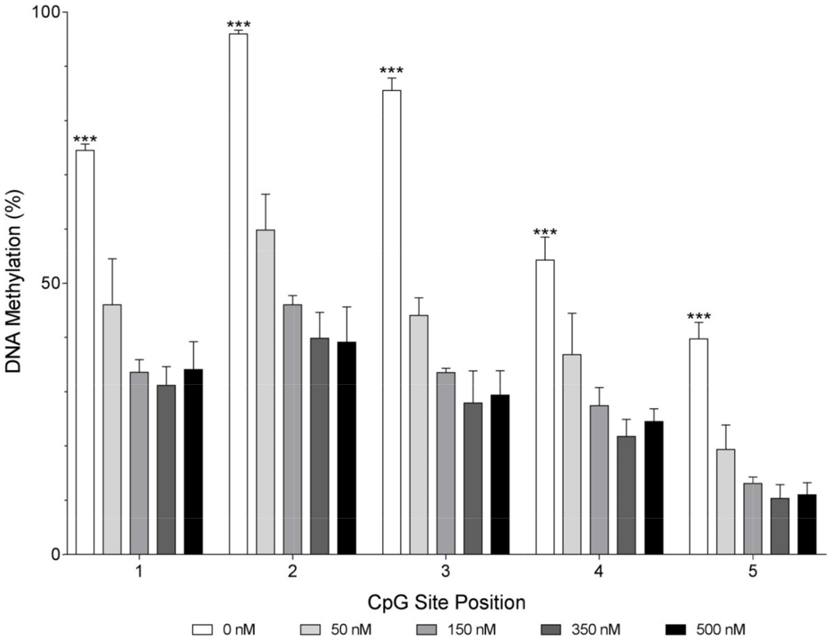

Supplementary Figure 8: Decitabine causes demethylation of both *EPOR* and *GATA2* in NALM-6 cells. (A) Methylation status of the 18 CpG sites of the *EPOR* promoter region in NALM-6 cells after treatment with 50 to 500 nM Decitabine compared to control. **(B)** Methylation status of the 5 CpG sites of the *GATA2* promoter region in NALM-6 cells after treatment with Decitabine compared to control. Expression values were corrected to 18S ribosomal RNA levels. Mean corrected Ct values (±SD) are shown. Statistical differences compared to the control (0 nM Decitabine) are indicated by * ($p < 0.05$), ** ($p < 0.01$), or *** ($p < 0.001$) were calculated with the two-way ANOVA with Holm-Sidak correction.

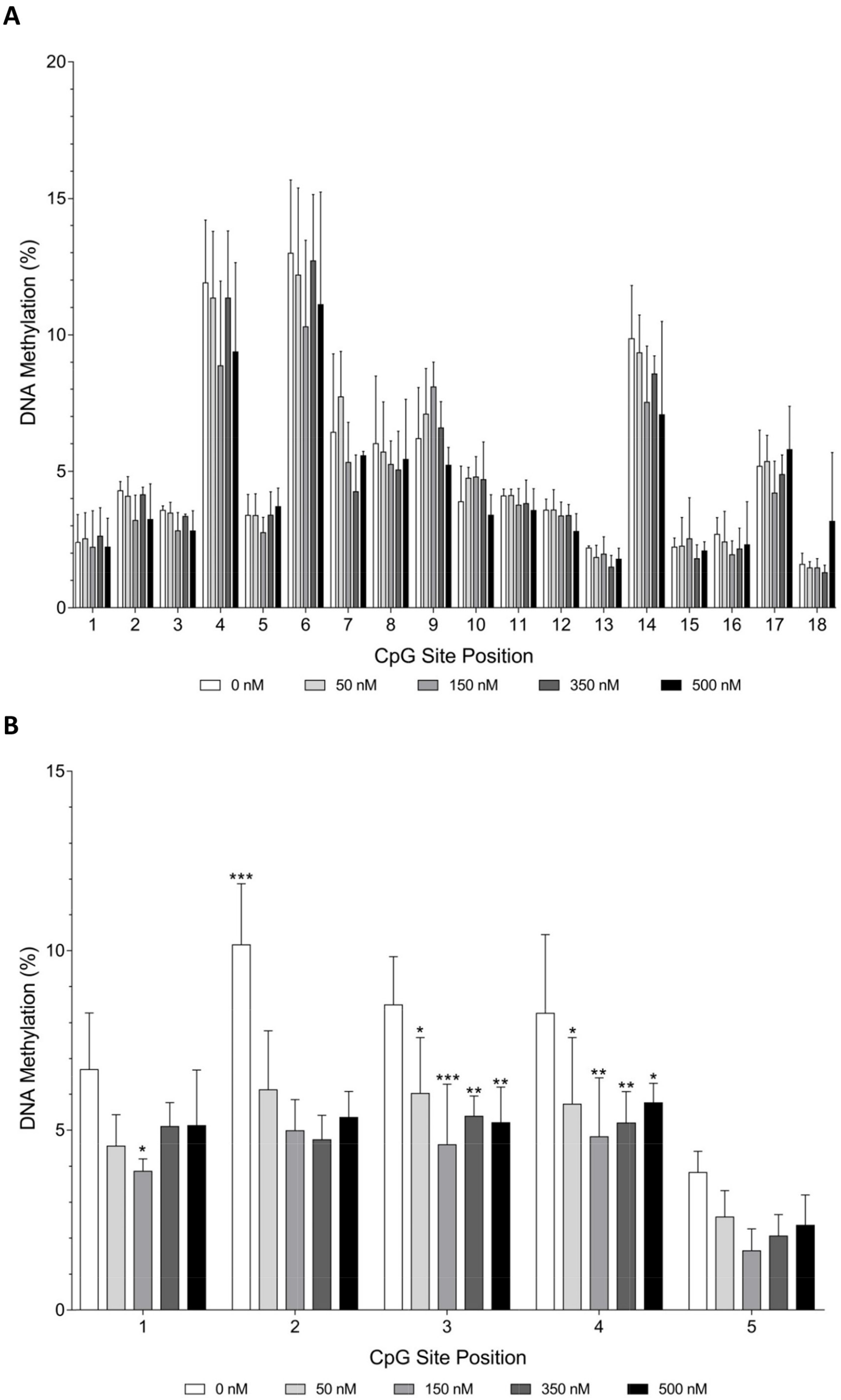

Supplementary Figure 9: Decitabine causes demethylation of *GATA2* but not *EPOR* in REH cells. **(A)** Methylation status of the 18 CpG sites of the *EPOR* promoter region in REH cells after treatment with 50 to 500 nM Decitabine compared to control. **(B)** Methylation status of the 5 CpG sites of the *GATA2* promoter region in REH cells after treatment with Decitabine. Statistical differences compared to the control (0 nM Decitabine) are indicated by * ($p < 0.05$), ** ($p < 0.01$), or *** ($p < 0.001$) were calculated with the two-way ANOVA with Holm-Sidak correction.

Supplementary Table 1: Base media and additives used for the culture of each cell line

Cell line	Media
**REH**	RPMI media (inc. L-glutamine; PAA), 100 U/ml penicillin (Invitrogen), 100 µg/ml streptomycin (Invitrogen), and 10% HI-FCS (Invitrogen)
**NALM-6**	RPMI media (inc. L-glutamine; PAA), 100 U/ml penicillin (Invitrogen), 100 µg/ml streptomycin (Invitrogen), and 10% HI-FCS (Invitrogen)
**UT-7**	MEMα media (inc. L-glutamine and nucleosides; PAA), 100 U/ml penicillin (Invitrogen), 100 µg/ml streptomycin (Invitrogen), 10% HI-FCS (Invitrogen), and 10 ng/ml human GM-CSF (PeproTech).

Supplementary Table 2: Primers for pyrosequencing assays to detect DNA methylation levels in the 5′ DNA of *EPOR* and *GATA2*

Pyrosequencing target	Forward primer	Reverse primer (* indicates 5′ biotinylation)	Sequencing primer (CpG sites are shown in brackets)
**GATA2**	TGGGGTGAGGG TTTTTTAGTG	*CCTCCCCCTCCCATTAACT	TTAGGAGAGAGTAGGGA
**EPOR (1st set)**	GTAAGTTATTTG TTTAGGGTTATA	*CCAACACTCAACCTAAATA	TGTTTAGGGTTATAAAGA (1-4)<br>TTTAAGAGGTTTTTGG (5-6)
**EPOR (2nd set)**	TTTATTTTTATTTA GGTTGAGTGT	*CCCAAATAATCCATAATACAA	TTATTTTTATTTAGGTTGAGTG (7-10)<br>GGAGTTTGGTAGGGAAG (11-13)<br>GTTAGGAGGAGGTAGT (14-16)<br>AAGGAGGGGTAGGAGT (17-18)

Multiple pyrosequencing PCRs and sequencing reactions were required to evaluate the 18 CpG sites in the *EPOR* 5′ DNA

Supplementary Table 3: Correlation between all the *EPOR* and *GATA1-6* probesets on the Affymetrix U133Plus2 array for the ALL with hyper-diploid karyotype and t(12:21) ALL patients in the MILE study [26].

See Supplementary File 1

Supplementary Table 4: MicroRNAs predicted to target either EPOR or GATA2 by both the Pareto Front software and in publicly available microRNA target databases

Predicted EPOR targeting microRNAs	Predicted GATA2 targeting microRNAs
hsa-miR-1200	hsa-miR-128
hsa-miR-1205	hsa-miR-132
hsa-miR-1246	hsa-miR-200b
hsa-miR-125a-5p	hsa-miR-200c
hsa-miR-125b	hsa-miR-212
hsa-miR-1274a	hsa-miR-25
hsa-miR-1274b	hsa-miR-27a
hsa-miR-1284	hsa-miR-27b
hsa-miR-188-5p	hsa-miR-32
hsa-miR-200b	hsa-miR-338-5p
hsa-miR-200c	hsa-miR-363
hsa-miR-217	hsa-miR-367
hsa-miR-296-3p	hsa-miR-409-3p
hsa-miR-330-5p	hsa-miR-429
hsa-miR-339-5p	hsa-miR-515-5p
hsa-miR-362-5p	hsa-miR-522
hsa-miR-365	hsa-miR-581
hsa-miR-429	hsa-miR-646
hsa-miR-509-3-5p	hsa-miR-650
hsa-miR-526b	hsa-miR-92a
hsa-miR-557	hsa-miR-92b
hsa-miR-587	
